# Wavelength-scale light concentrator made by direct 3D laser writing of polymer metamaterials

**DOI:** 10.1038/srep33627

**Published:** 2016-10-04

**Authors:** J. Moughames, S. Jradi, T. M. Chan, S. Akil, Y. Battie, A. En Naciri, Z. Herro, S. Guenneau, S. Enoch, L. Joly, J. Cousin, A. Bruyant

**Affiliations:** 1 Laboratoire de Nanotechnologie et d’Instrumentation Optique, ICD, CNRS UMR 6281, Université de Technologie de Troyes, 12 Rue Marie Curie CS42060, 10004 Troyes Cedex, France; 2 Laboratoire de Physique Appliquée, Université Libanaise, Faculté des Sciences II, Fanar, Liban; 3 Aix-Marseille Univ., CNRS, Centrale Marseille, Institut Fresnel UMR 7249, 13013 Marseille, France; 4 Laboratoire de Chimie et Physique, Université de Lorraine, 1 Bd Arago, 57070 Metz Technopôle, France; 5 Groupe de Spectrométrie Moléculaire et Atmosphérique GSMA, UMR CNRS 7331, Université de Reims, U.F.R. Sciences Exactes et Naturelles, Moulin de la Housse B.P. 1039, F-51687 Reims, France

## Abstract

We report on the realization of functional infrared light concentrators based on a thick layer of air-polymer metamaterial with controlled pore size gradients. The design features an optimum gradient index profile leading to light focusing in the Fresnel zone of the structures for two selected operating wavelength domains near 5.6 and 10.4 μm. The metamaterial which consists in a thick polymer containing air holes with diameters ranging from λ/20 to λ/8 is made using a 3D lithography technique based on the two-photon polymerization of a homemade photopolymer. Infrared imaging of the structures reveals a tight focusing for both structures with a maximum local intensity increase by a factor of 2.5 for a concentrator volume of 1.5 λ^3^, slightly limited by the residual absorption of the selected polymer. Such porous and flat metamaterial structures offer interesting perspectives to increase infrared detector performance at the pixel level for imaging or sensing applications.

Gradient index optics plays a major role in micro-optics and micro-photonics to efficiently manipulate light using compact and simple shape optics^1^,^2^,^3^,^4^,^5^,^6^. This attribute has driven a number of applications notably in fiber-optic communication, or optical medical devices where they can be used for low-aberration imaging in a space-effective way^7^,^8^,^9^. In the context of infrared detection and notably mid-infrared imaging, flat gradient index (GRIN) lenses represent an attractive solution to enhance the photometric performances by concentrating light onto ever smaller active areas. In fact, the ability to focus light directly at the pixel level is highly desirable to reduce the detector volume and its thermal noise, while having an optimal footprint and resolution. However, challenges still remain in the integration of light concentrators with dimensions compatible with the small detector size and pitch.

The interest in down-scaled GRIN lens was recently renewed in the track of metamaterials (MM) studies^10^,^11^,^12^,^13^,^14^ because such engineered structures offer unprecedented control over the achievable refractive index. In this context, compact designs of Metamaterial GRIN lens derived from transformation optics (TO) methods were reported^15^,^16^.

It was notably suggested to design a flat metamaterial layer analogous to the Maxwell fisheye and Luneburg’s lens for which the refractive index corresponds to the stereographic projection of a sphere on a plane^17^,^18^. MM made of toroidal inclusions were proposed to achieve the hyperbolic secant index profile^10^,^11^, known to produce stigmatic images^19^ and corresponding to the Mercator projection of a sphere on plane^17^,^18^. Based on these simulation results^10^, it appears that a few micrometer thick MM layer of small volume (in the order of λ^3^) can produce the required tight and efficient focusing in the mid-IR with a negligible footprint. Standard GRIN lenses of different materials with continuous index profile can be fabricated via different techniques such as Chemical Vapor Deposition (CVD)^20^, ion exchange^21^,^22^,^23^, thermal^24^ and UV polymerization^25^ but all these techniques typically lack both spatial resolution and sufficient refractive index contrast to scale down the lens size.

So far, MM GRIN lenses were realized and tested in the acoustic and microwave domains^26^,^27^,^28^. For shorter wavelengths, micro-scaled GRIN lenses with a soft, continuous, gradient index distribution in a polymer block were proposed^29^ by spatially tuning the cross-linking of the photopolymer under different irradiation conditions. However, the achievable contrast of refractive index measured in the visible is very low (~0.01) and no experimental demonstration of light focusing effect was shown. In the field of diffractive optics, flat lenses based on photonic crystals^30^ or blazed binary gratings^31^ were also proposed for imaging purpose. For such structures, the pitch is comparable or larger than the wavelength and their response is chromatic. Thus, the possible roads to achieve the fabrication of the proposed MM GRIN lenses in the infrared range (e.g. between 5–11 μm) with the required subwavelength discretization step and a sufficient index contrast still have to be explored.

In this work, the fabrication of functional IR MM GRIN lenses is carried out using a 3D lithography technique based on Two Photon Polymerization (TPP) of photopolymers. This technique is distinguished by its flexibility, swiftness to fabricate complex 3D microstructures, and its high spatial resolution able to produce minute details (<100 nm) over relatively large scale (>100 μm). In addition, for reasonably small concentrator thickness (e.g. <10 μm), a number of transparencies windows are present in the infrared range. Thus the microstructured polymer thick film was used as the final material without the need of further transfer processes. This makes TPP very suitable for direct realization and optimization of functional 3D MM concentrators prior more demanding conventional photolithography and etching processes. The possibility of rapid prototyping of 3D designs and testing makes it possible to adopt an iterative approach of simulation, fabrication and characterization.

## Results

### Infrared concentrator design

**Figure 1:**
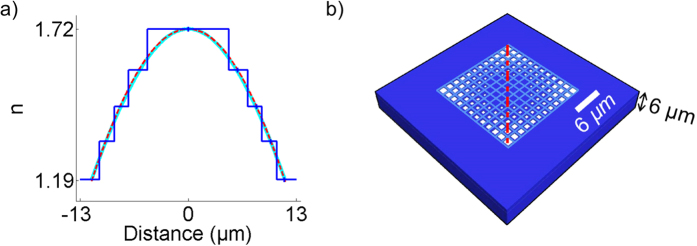
(**a**) Example of refractive index profiles given by the secant hyperbolic relation (in cyan); the derived expression (dashed red), and corresponding discretized refractive index (dark blue) along the diagonal profile shown in the device top view. (**b**) Top view of the device. The square holes sizes are position dependent.

In order to design the MM GRIN^32^,^33^ lens, shortly referred to as “MM concentrator”, our approach consists in creating an effective radial refractive index profile *n(r)* by introducing an adequate concentration of subwavelength holes in the polymer block (cf. Fig. 1(a)). The ideal hyperbolic secant distribution previously proposed to focus light^34^,^35^,^36^ with MM is expressed as:


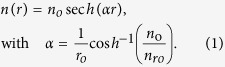


where, *n*_*o*_ is the refractive index of the bulk polymer at the concentrator center at *r* = 0, and *n*_*ro*_ is the minimum refractive index of the porous polymer at the concentrator edge at *r* = *r*_*o*_. For an incident parallel beam, such profile is known for long^19^ to produce repeated astigmatic focus spots within the graded index material, that are characterized by a pitch length 4*f* = 2*π*/*α*. The position of the first spot with respect to the first interface is equal to *f* and can be calculated considering the pixel size (*r*_*o*_), and the achievable refractive index ratio. This quarter pitch distance corresponds to the flat lens thickness needed to obtain the best focus exactly at the second interface. However, this thickness can be further decreased (*t* < *f*) to produce a spot in the external air medium, in the vicinity of the second interface. The aberrations are minimal as long as the focus spot is kept within a subwavelength distance from the interface. While it is interesting to work with the smallest thickness *t* to speed up and facilitate the fabrication process, simulations show that a minimum thickness is required for a given pixel size (e.g. 2*r*_*o*_ ~ two wavelengths) to achieve a correct operation. In the case of wavelength-scale concentrator, the paraxial formula giving the working distance from the second interface^37^ strongly overestimates the spot position for such wavelength-scale structures and electromagnetic simulation should be carried out to determine more precisely the short working distance for different *t*. An alternative analytic estimation was derived by neglecting the impact of the radial propagation in the material on the phase of the rays. By considering only the longitudinal optical path *l*(*r*) = *n*(*r*)*t, needed *t*o transform an input plane wave front into an output spherical wave of the form exp (*ik*(|***r*** − ***r***_***f***_|)), focusing in ***r***_*****f*****_ at a working distance *WD* from the interface, the following relations are then obtained:


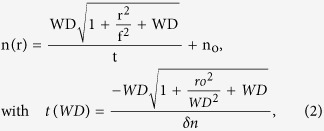


where *k* = 2*π/λ* is the free space wave number. Using the latter relation, *WD* and *r*_*o*_ can be freely chosen for an achievable index contrast *δ*n = *n*_*ro*_ − *n*_*o*_, thus determining the thickness t and the required refractive index profile *n*(*r*). The Fig. 1(a) exemplifies the refractive index profiles similarity given by the two approaches for typical parameters used in this work. The index profiles are almost identical within the small concentrator region (*r* < *r*_*o*_). While the above formula provides a coarse estimation for the wavelength-scale concentrators’ parameters (*t*, *n*(*r*)), the working distance revealed by electromagnetic simulations tends to be much smaller than the input value WD when *t* is smaller or approximately equals to the wavelength. These aspects are discussed in the Supplemental Material (Supplemental Fig. S1).

### Effective index determination

In order to achieve the nearly equivalent distributions given by eqs 1 or 2, the porosity of the material is engineered with the inclusion of subwavelength air holes inside the material, as shown in Fig. 1(b). The actual size of the square holes is deduced from the required effective permittivity ***ε***_e_ = *n*^2^(*r*) using the effective medium described by the Maxwell-Garnett equation^38^


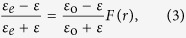


where ***ε***_0_ = 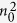 and ***ε*** = 1 are the permittivities of the resist and background (air), respectively, and *F*(*r*) is the filling fraction equals to the cross-sectional areas of the material (***ε***_0_) normalized by the surface of the cells visible in Fig. 1(b).

The negative liquid photoresist is a mixture made from Pentaerythritol triacrylate (PETA) which has been widely used as polymerizable monomer to fabricate micro-optical elements^39^,^40^,^41^,^42^, and 2.4% of Irgacure 819 (Irg 819) as photoinitiator^43^.

**Figure 2:**
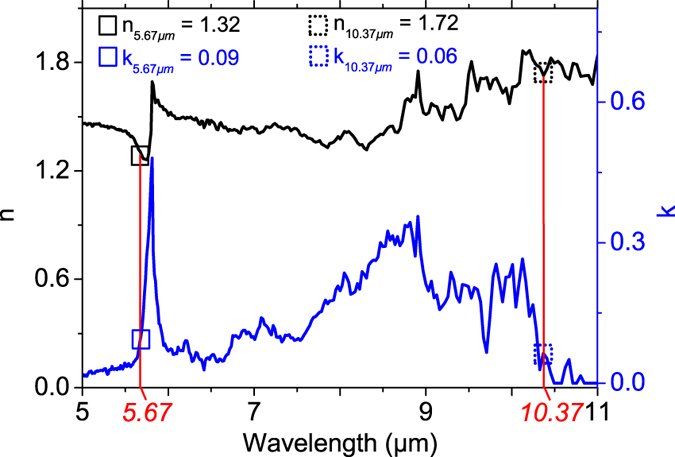
Real and imaginary parts of the polymer refractive index obtained from infrared ellipsometric measurement, by using a wavelength by wavelength inversion method^44^.

To determine the possible spectral range of operation, the transmission of the considered polymer was measured by Fourier transform infrared spectroscopy (FTIR) technique. The infrared refractive indices of the bulk polymerized resist were also estimated by spectroscopic ellipsometry, in order to determine for each wavelength^44^ the maximum refractive index available n_o_ (without porosity). The result is shown in Fig. 2. For the actual design, two operating wavelength ranges near 5.6 and 10.4 μm were selected close to the emission lines of our available laser sources. For these wavelengths, the real refractive index *n*_*o*_ is found to be close to 1.32 and 1.72, respectively.

### MM concentrator optimization

Small concentrator sizes compatible with a possible integration at a pixel level were selected. Concentrator areas slightly smaller than 3λ^2^ (11 μm ∗ 11 μm and 18 μm ∗ 18 μm, respectively) were hence designed, with short working distances *f* close to *λ* (1 and 2.1 μm, respectively). For such parameters and considering the material absorption an optimum metamaterial layer thicknesses were found by simulation to be about 3 and 6 microns, respectively, resulting in concentrator volumes of about 1.5λ^3^. The width of the subwavelength square holes radially increases from 240 nm to 600 nm for the first concentrator and from 450 nm to 1.2 μm for the second one (i.e. from about λ/20 to λ/8).

The 3D photo-polymerization was performed using a femtosecond TPP-based technique from Nanoscribe. Such Direct Laser Writing (DLW) was used extensively in the past few years to realize nearly arbitrary 3D patterning^45^,^46^. In order to obtain the required high spatial resolution, we have studied the behavior of our photoresist by measuring the polymer linewidth obtained for different scan speeds. Linewidth down to 75 nm were obtained and are reported in Supplemental Information (cf. Supplemental Fig. S2). For both structures, a constant writing speed of *v* = 75 μm/s was found to be satisfactory compromise between resolution and speed to process the whole structure in about 4 hours.

**Figure 3:**
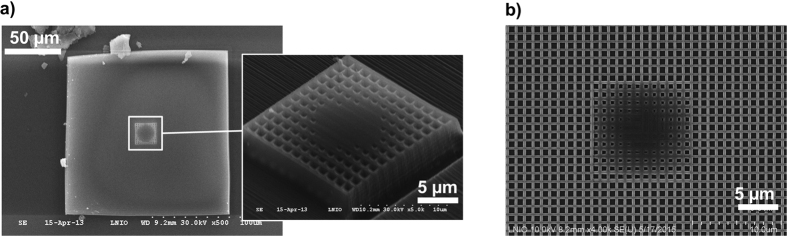
(**a**) SEM top view of the MM concentrator designed for a wavelength of 10.4 μm integrated in a 150 μm thin polymer block. The inset is a SEM side view of MM concentrator; (**b**) SEM top view of the MM concentrator designed for 5.6 μm.

Figure 3(a,b) show the SEM images of the experimental realizations having dimensions optimized for two different wavelengths, i.e. 10.4 μm and 5.6 μm respectively. The first structure was made with the above mentioned scan speed at a laser power of 7 mW. In order to minimize the diffraction by the structure edge, the concentrator was integrated in a thin square block of polymer of 150 μm size. The second one was written at the same speed but at a slightly smaller light power of 6 mW to achieve smaller dimensions (the writing linewidth is about 80 nm). In that case, an impedance matching was done by embedding the structure in a 75 μm wide grid 0.6 × 0.6 μm^2^ square holes in order to obtain the same effective refractive index as this of the concentrator edge.

**Figure 4:**
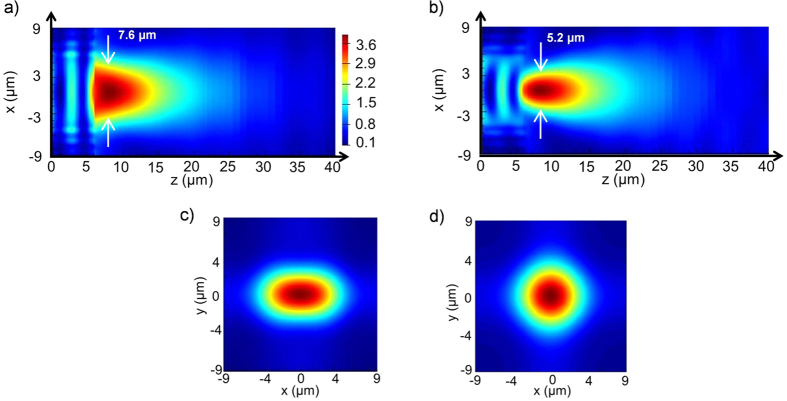
3D Finite Difference Time Domains (FDTD) simulation of the MM concentrator spot (energy density enhancement) at λ = 10.4 μm for the 6 μm high concentrator. (**a,b**) Axial cross-sections in the (x, z) and (y, z) plans respectively for an incident plane wave polarization along x. (**c**) Simulation in the (x, y) transverse plan at the focus maximum for the same incident plane wave. (**d**) Simulation in the (x, y) transverse plan at the focus maximum for a circular polarization, calculated by averaging the energy densities of a x-polarized and a y-polarized plane wave.

Figure 4 shows a 3D electromagnetic simulation of the MM structure designed at 10.4 μm, illuminated from the bottom by a plane wave polarized along the *x* axis direction. The field intensity is normalized by the field intensity obtained without the MM lens. The spot size obtained for the discretized profile is slightly smaller than *λ* (7.6 and 5.2 μm measured along the *x* and *y* axis respectively at Full Width Half Maximum). In fact, compared to simulations performed on a concentrator with a continuous index distributions (shown in the Supplemental Fig. S3), the working distance is found to be shorter (2.1 μm *vs* 7.5 μm) and the focusing is a bit tighter. This can be understood by the fact that the refractive index profile tends to be higher in the center of the MM refractive index distribution due to the rather large discretization step. The discretization also introduces some anisotropy between the transverse and axial planes which is not accounted by the isotropic effective medium theory employed.

### Infrared characterization

**Figure 5:**
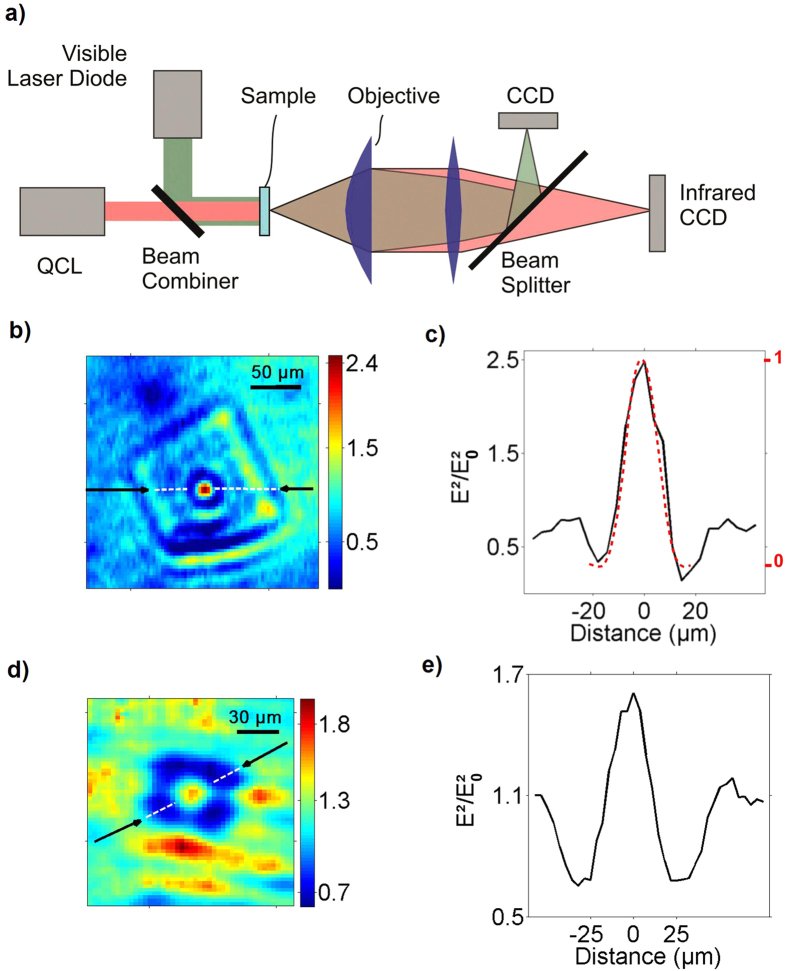
(**a**) Schematic representation of the experimental setup. (**b**) IR CCD image of the larger concentrator at a wavelength of λ = 10.4 μm. The image is normalized by the intensity recorded without polymer structure. (**c**) Related intensity profile measure along the segment marked by black arrows in (**b**). The profile of the point spread function of the objective lens is shown in dashed red. (**d**) Normalized IR CCD image of the smaller concentrator at a wavelength of λ = 5.67 μm. (**e**) Related intensity profile measured along the segment marked by the black arrows in (**d**).

The focusing effect of the fabricated structures was imaged with the IR set-up shown in Fig. 5(a) using an IR camera assisted by a visible light microscope. The later was adjusted for each wavelength to image the same object plane than the IR camera by simultaneously imaging a bright point scatterer. The measurements were done in transmission on each structure with two available quantum cascade laser sources (5.67 μm or 10.37 μm). A ZnSe aspheric lens with a numerical aperture *NA* = 0.4 was used as an objective lens leading to a theoretical Abbe resolution limit of about *9* and *16* μm, respectively.

**Figure 6:**
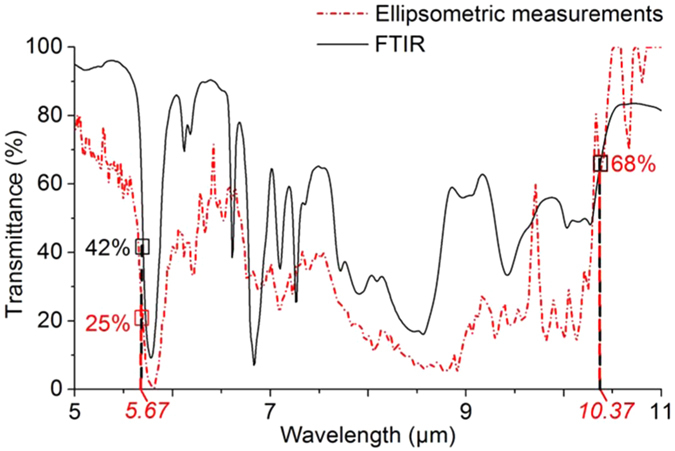
FTIR and ellipsometric measurements carried out on a polymerized resist film with a thickness of approximately 6 μm. The two spectra allow us to identify transparency windows in the IR domain. The selected IR wavelengths are shown in red.

The obtained IR images in Fig. 5(b,d) and profiles in Fig. 5(c,e) reveal the presence of a focus spot in the immediate vicinity of MM lens. One can see from these far-field images that the first MM lens designed for the 10.37 μm exhibits the expected, mostly diffraction limited spot as can be judged by comparing the experimental profile and the point spread function of the objective lens shown in Fig. 5(c). In comparison, the smallest MM lens designed for the 5.67 μm presents a more modest confinement mainly due to the smaller *n*_*o*_ value (about 1.32 *vs.* 1.72) and index contrast (about 0.23 *vs.* 0.53). On both images, the spots are surrounded by a dark region spreading over zones where the polymer fraction is higher. This observation is attributed to the remaining absorption of the polymer. This absorption was confirmed by the FTIR spectrum shown in Fig. 6 of a 6 μm layer of UV that shows a transmittance of 68% at 10.37 μm. A smaller light transmittance was observed at 5.67 μm due to the proximity of a sharp absorption peak. While the resist formulation used for the transmittance measurement is the same than the concentrator material, caution must be exercised in the determination of the real absorption value since the polymerization process for the UV-cured resist is not the same as the TPP induced by the DLW. Experimental intensity enhancement of 2.5 and 1.5 are obtained while enhancement factor of 3.6 and 2.3 are expected from 3D simulations taking into account the polymer losses experimentally measured for the two different wavelengths. We also note the presence of some unwanted scattering near the edges of the structures producing relative intensity peaks of about 1.5. The experimental enhancement factors were estimated by dividing the image of the focus spot by the signal measured with the same microscope in a zone on the sample surface where no polymer is present. Therefore, the measured enhancement in the concentrator center is also lowered by the polymer absorption as for the simulations.

In summary, interesting performances were achieved by the larger concentrator, where the experimental 2.5 factor obtained here could represent approximately the same detector volume reduction providing ideally a similar thermal noise reduction. While the spot where imaged at precise wavelengths, we point out that the focusing properties of the MM are not strongly chromatic within a transparent region. Also, the adopted 2D design makes it compatible with a possible reactive ion etching transfer within a robust material such as Si that should provide stronger enhancement due to the higher *n*_*o*_ and achievable δn value.

## Conclusion

In conclusion, we have designed, simulated, fabricated and investigated IR concentrators at two infrared operating wavelengths near 10.4 μm and 5.6 μm. The general design rule has been described for the fabrication of these MM based GRIN lenses that can find a natural application for focusing light at the pixel level inside or outside the IR domain. The structures were made by direct laser writing of a homemade triacrylate polymer featuring high spatial resolution using the two photon absorption technique. The results showed a clear light focusing through the wavelength-scale concentrators (~1.5*λ*^3^) with a maximum intensity increase by a factor of 2.5 for the larger concentrator, and a lesser increase for the smaller concentrator due to reduced refractive index contrast and residual polymer absorption. Considering that the MM concentrator slabs are invariant in the axial direction and not too thick, it is foreseen that similar structures could be made using other high refractive index materials such as silicon and more conventional 2D based lithography coupled with deep reactive ion etching. The developed MM lens also offers an interesting flat platform in its center, where light can be concentrated, for additional light manipulation such as plasmonic lensing.

## Methods

### Measurement of the transparency windows

Transmission FTIR was performed in order to detect the possible operational, transparent, zones of the resist in the IR domain. To perform the measurement, the resist was tightly sandwiched between two NaCl substrates with a spacing of *6* μm, thus defining the polymer slab thickness. The FTIR transmittance spectrum is shown in Fig. 6 (black curve). The transparency windows identified with the FTIR technique are qualitatively accounted for by the transmittance calculated from the extinction index values extracted by the spectroscopic ellipsometry technique (red curve).

### Spectroscopic ellipsometry

The measurement was performed on the polymer film deposited on Si substrate by using a home-built rotating polarizer ellipsometer coupled with a FTIR. The optical constants are deduced from the ellipsometric parameters tanΨ and cos Δ assuming a simple system composed of a film deposited on a silicon substrate. The film thickness is first estimated from ellipsometric measurements in the visible spectral range by using a phase modulated ellipsometer (Horiba Jobin Yvon, UVISEL). Then, a wavelength by wavelength inversion^42^ is used to determine the complex refractive index of the PETA-based polymer.

### 3D Direct laser writing

The femtosecond laser beam (repetition rate: *100* *MHz*, wavelength: *λ*_*DLW*_ = 780 nm, pulse duration: <*140 fs*) is focused into the sample by a high numerical aperture objective lens (NA = 1.4). The TPP fabrication process is monitored in real time via the same objective lens by a CCD camera; the polymerized structure being visible due to the induced changes in the refractive index of photosensitive material. Because most of the IR-transparent substrates are too thick or opaque at the fabrication wavelength, the liquid resist was sandwiched between a thin glass coverslip (170 μm thickness) transparent at *λ*_DLW_, and a thick substrate of NaCl (transparent in the IR domain) separated from the coverslip by a 17 μm spacer. The writing was then made onto the NaCl substrate through the glass coverslip. Following the exposure, the sample cell was rinsed in isopropanol during 30 minutes to remove the unexposed resist. Details regarding the achieved spatial resolution are given in Supplemental Information.

## Additional Information

**How to cite this article**: Moughames, J. *et al*. Wavelength-scale light concentrator made by direct 3D laser writing of polymer metamaterials. *Sci. Rep.* **6**, 33627; doi: 10.1038/srep33627 (2016).

## Supplementary Material

Supplementary Information

## References

[b1] Bähr J., Brenner K.-H., Sinzinger S., Spick T. & Testorf M. Index-distributed planar microlenses for three-dimensional micro-optics fabricated by silver-sodium ion exchange in BGG35 substrates. Applied optics 33, 5919–5924 (1994). 20935998 10.1364/AO.33.005919

[b2] Delage A. . Graded-index coupler for microphotonic SOI waveguides. Photonics North 204–212 (2004).

[b3] Janz S. . Silicon-based integrated optics: Waveguide technology to microphotonics. MRS Proc. 832, F1. 1 (2004).

[b4] Janz S. . Microphotonic elements for integration on the silicon–on–insulator waveguide platform. IEEE Journal 12, 1402–1415 (2006).

[b5] Kress B. C. & Meyrueis P. Of referencing In Applied digital optics: from micro-optics to nanophotonics (Wiley, 2009).

[b6] Yulin L., Tonghai L., Guohua J., Baowen H., Junmin H. & Lili W. Research on micro-optical lenses fabrication technology. Optik-International Journal for Light and Electron Optics 118, 395–401 (2007).

[b7] Bird D. & Gu M. Two-photon fluorescence endoscopy with a micro-optic scanning head. Optics Letters 28, 1552–1554 (2003). 12956376 10.1364/ol.28.001552

[b8] Cheben P., Xu D. X., Janz S. & Densmore A. Subwavelength waveguide grating for mode conversion and light coupling in integrated optics. Optics Express 14, 4695–4702 (2006). 19516625 10.1364/oe.14.004695

[b9] Lee W. M. & Yun S. H. Adaptive aberration correction of GRIN lenses for confocal endomicroscopy. Optics Letters 36, 4608–4610 (2011). 22139258 10.1364/OL.36.004608 PMC3461260

[b10] Chang T. M., Guenneau S., Hazart J. & Enoch S. Focussing light through a stack of toroidal channels in PMMA. Optics express 19, 16154–16159 (2011). 21934978 10.1364/OE.19.016154

[b11] Chang T. M., Dupont G., Enoch S. & Guenneau S. Enhanced control of light and sound trajectories with three-dimensional gradient index lenses. New Journal of Physics 14, 035011 (2012).

[b12] Rahm M., Schurig D., Roberts D. A., Cummer S. A., Smith D. R. & Pendry J. B. Design of electromagnetic cloaks and concentrators using form-invariant coordinate transformations of Maxwell’s equations. Photonics and Nanostructures-fundamentals and Applications 6, 87–95 (2008).

[b13] Yang R., Wang Z., Su H., Yang Y. & Lei Z. Bi-functional Fresnel zone plate from transformation optics. Journal of Optics 17, 075602 (2015).

[b14] Yi J., Burokur S. N. & De Lustrac A. Conceptual design of a beam steering lens through transformation electromagnetics. Optics Express 23, 12942–12951 (2015). 26074547 10.1364/OE.23.012942

[b15] Kang M., Feng T., Wang H. T. & Li J. Wave front engineering from an array of thin aperture antennas. Optics Express 20, 15882–15890 (2012). 22772278 10.1364/OE.20.015882

[b16] Yu N. . Light propagation with phase discontinuities: generalized laws of reflection and refraction. Science 334, 333–337 (2011). 21885733 10.1126/science.1210713

[b17] Philbin T. & Leonhardt U. Of referencing In Geometry and Light: The Science of Invisibility. (Courier Corporation, 2010).

[b18] Mantel K., Bachstein D. & Peschel U. Perfect imaging of hypersurfaces via transformation optics. Optics Letters 36, 199–201 (2011). 21263499 10.1364/OL.36.000199

[b19] Fletcher A., Murphy T. & Young A. Solutions of two optical problems. Proceedings of the Royal Society 223, 216–225 (1954).

[b20] Pickering M. A., Taylor R. L. & Moore D. T. Gradient infrared optical material prepared by a chemical vapor deposition process. Applied optics 25, 3364–3372 (1986). 18235630 10.1364/ao.25.003364

[b21] Sinai P. Correction of optical aberrations by neutron irradiation. Applied optics 10, 99–104 (1971). 20094398 10.1364/AO.10.000099

[b22] Ohmi S., Sakai H., Asahara Y., Nakayama S., Yoneda Y. & Izumitani T. Gradient-index rod lens made by a double ion-exchange process. Applied optics 27, 496–499 (1988). 20523629 10.1364/AO.27.000496

[b23] Messerschmidt B., Possner T. & Goering R. Colorless gradient-index cylindrical lenses with high numerical apertures produced by silver-ion exchange. Applied optics 34, 7825–7830 (1995). 21068874 10.1364/AO.34.007825

[b24] Liu J. H., Yang P. C. & Chiu Y. H. Fabrication of high performance, gradient refractive index plastic rods with surfmer cluster stabilized nanoparticles. Journal of Polymer Science Part A: Polymer Chemistry 44, 5933–5942 (2006).

[b25] Jahromi M. N. & Liu J. H. Gel effects on the fabrication of gradient refractive index plastic rods via energy-controlled polymerization. Journal of the Taiwan Institute of Chemical Engineers 43, 301–305 (2012).

[b26] Torrent D. & Sanchez–Dehesa J. Acoustic metamaterials for new two-dimensional sonic devices. New journal of physics 9, 323 (2007).

[b27] Allen J. W. & Wu B. I. Design and fabrication of an RF GRIN lens using 3D printing technology. SPIE OPTO 86240V (2013).

[b28] Liang M., Ng W. R., Chang K., Gbele K., Gehm M. E. & Xin H. A 3-D Luneburg lens antenna fabricated by polymer jetting rapid prototyping. IEEE Transactions on Antennas and Propagation 62, 1799–1807 (2014).

[b29] Zukauskas A., Matulaitiene I., Paipulas D., Niaura G., Malinauskas M. & Gadonas R. Tuning the refractive index in 3D direct laser writing lithography: towards GRIN microoptics. Laser & Photonics Reviews 9, 706–712 (2015).

[b30] Maigyte L. . Flat lensing in the visible frequency range by woodpile photonic crystals. Optics letters 38, 2376–2378 (2013). 23939053 10.1364/OL.38.002376

[b31] Lee M. S. L., Lalanne P., Rodier J. C., Chavel P., Cambril E. & Chen Y. Imaging with blazed-binary diffractive elements. Journal of Optics A: Pure and Applied Optics 4, S119 (2002).

[b32] Liu J. H. & Chiu Y. H. Process equipped with a sloped UV lamp for the fabrication of gradient-refractive-index lenses. Optics letters 34, 1393–1395 (2009). 19412283 10.1364/ol.34.001393

[b33] Moore D. T. Gradient-index optics: a review. Applied Optics 19, 1035–1038 (1980). 20220980 10.1364/AO.19.001035

[b34] Baer E., Hiltner P. A. & Shirk J. S. Multilayer polymer gradient index (GRIN) lenses. US Patent No 7,002,754 (2006).

[b35] Climente A., Torrent D. & Sanchez-Dehesa J. Sound focusing by gradient index sonic lenses. Applied Physics Letters 97, 104103 (2010).

[b36] Lin S. C. S., Huang T. J., Sun J. H. & Wu T. T. Gradient-index phononic crystals. Physical Review B 79, 094302 (2009).

[b37] Kapron F. P. Geometrical optics of parabolic index–gradient cylindrical lenses. JOSA 60, 1433–1436 (1970).

[b38] Landauer R. Electrical conductivity in inhomogeneous media. AIP Publishing 40, 2–45 (1978).

[b39] Jradi S., Soppera O., Lougnot D. J., Bachelot R. & Royer P. Tailoring the geometry of polymer tips on the end of optical fibers via control of physico-chemical parameters. Optical Materials 31, 640–646 (2009).

[b40] Zeng X. . Integration of polymer microlens array at fiber bundle extremity by photopolymerization. Optics Express 19, 4805–4814 (2011). 21445116 10.1364/OE.19.004805

[b41] Guattari F. . Balanced homodyne detection of Bragg microholograms in photopolymer for data storage. Optics Express 15, 2234–2243 (2007). 19532458 10.1364/oe.15.002234

[b42] Zheng S. . Rapid fabrication of micro-nanometric tapered fiber lens and characterization by a novel scanning optical microscope with submicron resolution. Optics Express 21, 30–38 (2013). 23388893 10.1364/OE.21.000030

[b43] Zhou X. . Two-Color Single Hybrid Plasmonic Nanoemitters with Real Time Switchable Dominant Emission Wavelength. Nano Letters 15, 7458–7466 (2015). 26437118 10.1021/acs.nanolett.5b02962

[b44] Schubert M. Of referencing In Infrared ellipsometry on semiconductor layer structures: phonons, plasmons, and polaritons (Springer Science & Business Media 2004).

[b45] Kawata S. & Sun H. B. Two-photon photopolymerization as a tool for making micro-devices. Applied Surface Science 208, 153–158 (2003).

[b46] Von Freymann G. . Three-Dimensional Nanostructures for Photonics. Advanced Functional Materials 20, 1038–1052 (2010).

