# Supplementary material for: Wavelength-scale light concentrator made by direct 3D laser writing of polymer metamaterials
Source: Sci Rep. 2016 Oct 4;6:33627. doi: 10.1038/srep33627 (PMC5048423; doi:10.1038/srep33627)
Supplement: Supplementary Information [file srep33627-s1.pdf]

Supplemental information

Appendix to

Wavelength-scale light concentrator made by direct 3D laser writing of polymer metamaterials

J. Moughames[1,2], S. Jradi[1], T.M. Chan[3], S. Akil[4], Y. Battie[4], A. En Naciri[4], Z. Herro[2], S. Guenneau[3], S. Enoch[3], L. Joly[5], J. Cousin[5] & A. Bruyant[1]

[1] Laboratoire de Nanotechnologie et d'Instrumentation Optique, ICD, CNRS UMR 6281, Université de Technologie de Troyes, 12 Rue Marie Curie CS42060, 10004 Troyes Cedex, France. [2] Laboratoire de Physique Appliquée, Université Libanaise, Faculté des Sciences II, Fanar, Liban. [3] Aix-Marseille Université, CNRS, Centrale Marseille, Institut Fresnel UMR 7249, 13013 Marseille, France. [4] Laboratoire de Chimie et Physique, Université de Lorraine, 1 Bd Arago, 57070 Metz Technopôle, France. [5] Groupe de Spectrométrie Moléculaire et Atmosphérique GSMA, UMR CNRS 7331, Université de Reims, U.F.R. Sciences Exactes et Naturelles, Moulin de la Housse B.P. 1039, F-51687 Reims, France. Correspondence and requests for materials should be addressed to J.M. (email: johnny.moughames@utt.fr) or S.J. (email: safi.jradi@utt.fr) or A.B. (email: aurelien.bruyant@utt.fr).

Working distance of the light concentrators with continuous refractive index

Radial lens having the refractive index distribution given by eq. 1.1 are perfectly stigmatic for an incident parallel beam when the focusing occurs inside the material, i.e. for thicknesses t higher or equal to the quarter pitch length f. When t is smaller than f, the light is focused at a distance WD (working distance) after the lens-air interface. In such cases, the aberrations typically increase with increasing WD. For paraxial rays, the WD value was derived as a function of t considering a parabolic approximation [31]:

$$WD^{-1} = n_o \alpha \tan (t\alpha) \qquad \text{(S1.1)}$$

When considering MM concentrators with rather large δn values and wavelength-scale dimensions, this *WD(t)* value tends to strongly overestimate the position of the maximum field intensity.

Fig. S1 (a) and (b) exemplify theoretical and simulated WD for small GRIN lenses with fixed parameters ($\lambda=10.5\ \mu m$, $n_o=1.6$, $\delta n=0.22$). The WD as a function of the lens thickness according to eq. (S1.1) is plotted and compared to 2D electromagnetic simulation results.

In **Fig. S1** (a), the considered lenses are larger ($r_o=5\lambda$ and 2.5λ) with an identical secant hyperbolic distribution given by eq. (1.1) and characterized by a contrast $\delta n=0.22$ defined in $r=2.5\lambda$. The relation (S1.1) gives a reasonable account of the WD distance decrease induced by the smaller size, while still overestimating the WD for short t where WD is expected much larger. We note that large WD values are not of practical interest since the aberration becomes quickly too large resulting in a small field exaltation.

In **Fig. S1** (b) the sizes of lenses are smaller (r_o close to 2λ and λ) with the same index distribution characterized by a contrast $\delta n=0.22$ defined near $r=\lambda$. For such small lenses, the standard equation mostly fails to predict the maxima positions. The relation (S1.1) works rather well for the larger one but fails to give a precise focus spot position for the smallest lens especially for small thickness. In the circled region, where the geometrical models break down, we note that the WD value is kept small and simulations (not shown here) show that a reasonable exaltation factor is maintained unless very small t values are considered (e.g. $< \lambda/2$). This region can be targeted to produce wavelength-scale or slightly subwavelength light concentrators.

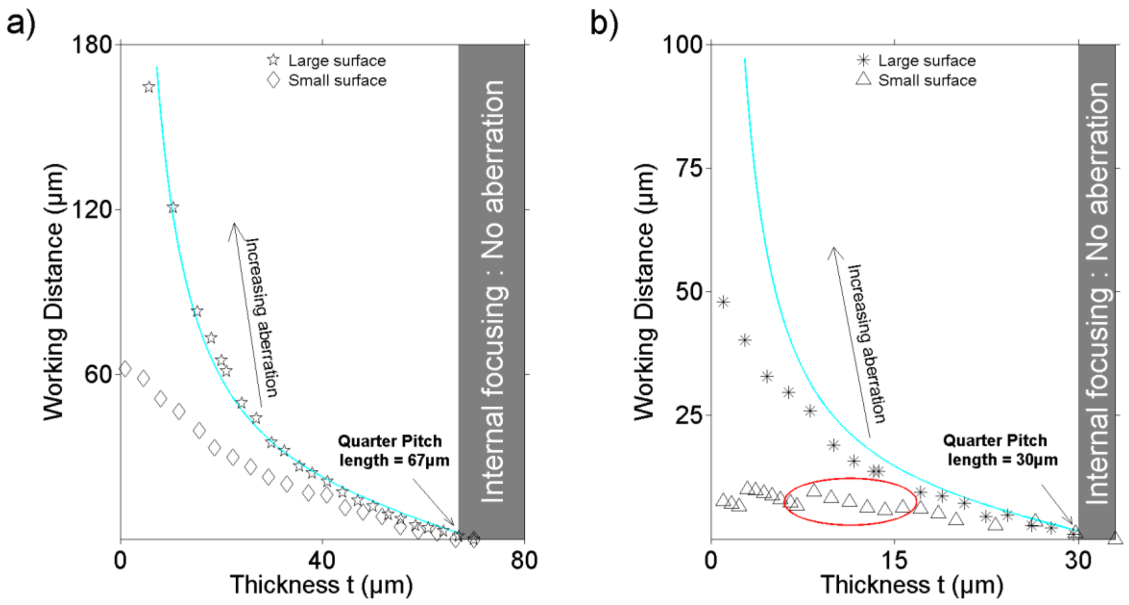

Fig. S1: Analytic and FDTD simulated working distances as a function of the lens thickness for (a) small concentrators: $r_o \approx 5\lambda$ and 2.5λ and (b) smaller concentrators $r_o \approx 2\lambda$ and λ. The analytic curves for *WD* (blue solid line) are given by eq. S1.1. FDTD-retrieved values appear as markers, the upper one (stars) corresponding to the larger lenses. The simulation parameters are $\lambda=10.4\ \mu m$, $n_o=1.6$, and $\delta n=0.22$ defined in $r=2.5\lambda$ in (a) and $r=\lambda$ in (b).

Comparison between continuous and discretized concentrator

In this section we give an estimation of the impact of the discretization on the field concentration compared to a continuous profile. In **Fig. S2**, 2D simulations show that continuous and discretized profiles give a similar pitch length if we consider an infinite concentrator along the *z* direction. The Maxwell-Garnett approximation seems to account quite precisely for the refractive index distribution of the discretized material in this 2D case, where the filling fraction *F(x)* corresponds to the ratio of the material and cell lengths along the *x* direction.

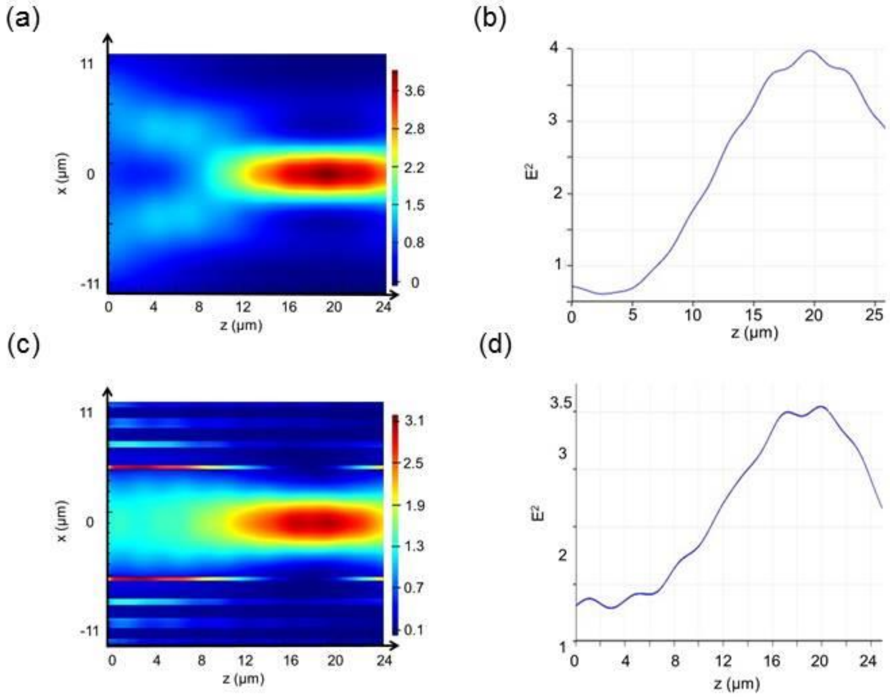

Fig. S2: 2D FDTD simulation of an infinite concentrator (a perfectly matched layer is added on the right to avoid reflection), for a continuous profile (a-b) and a discretized profile (c-d). The incident plane wave is polarized along the *x* direction.

If we consider a finite 3D discretized light concentrator, the agreement is more qualitative, showing the limit of the isotropic Maxwell Garnett approximation to account for the final system performance. The discrepancy can be judged from **Fig S3.** However, while the field enhancement is lower for a discretized structure we see that field turns to be even more confined near the interface.

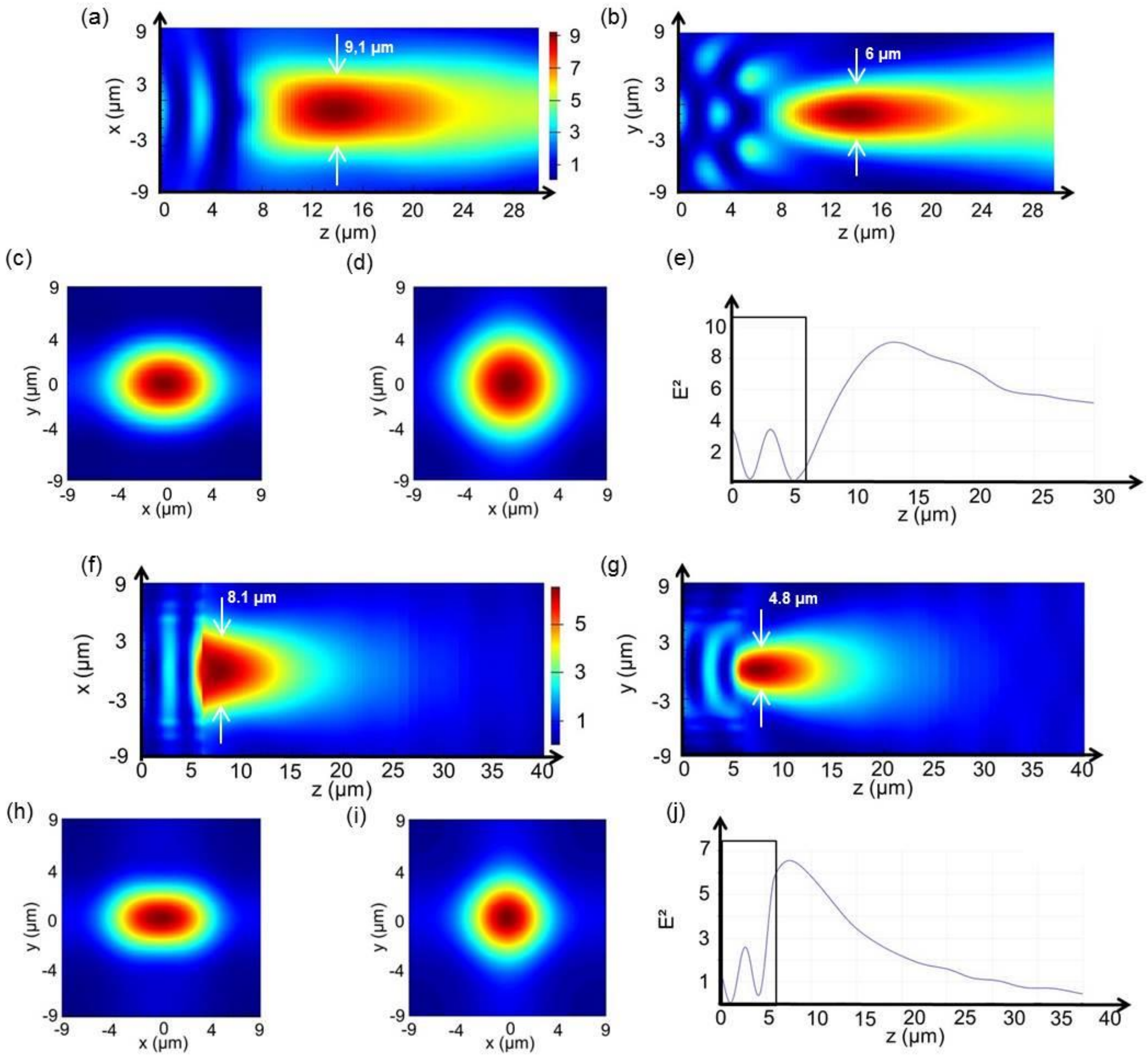

Fig. S3: 3D FDTD simulation of a lossless 3D MM concentrator spot (energy density) at $\lambda=10.4\ \mu m$ for the continuous profile **(a-e)** and the discretized profile **(f-j)**. The polarization of the incoming plane wave is along *x*. **(a-b)** Cross-section showing the focus spot in the *(x,z)* and *(y,z)* axial planes respectively. **(c)** Cross-section in the transverse *(x,y)* plane at the maximum position for *x*-polarized incident plane wave. **(d)** Energy density profiles along the optical axis: the first *6 µm* correspond to the MM layer, the spot maximum is approximately at 7.5 µm from the MM/air interface. **(e)** Cross-section in the transverse (x,y) plane at the maximum position for a circular polarization. **(f-i)** Cross-sections similar to (a-d) for the discretized concentrator. **(j)** Energy density profile along the optical axis: the spot maximum is approximately at *1.6 µm* from the MM/air interface.

Fabrication details: linewidths

Matrix of lines where written at various laser powers and scan speeds in order to determine the optimal writing parameters to achieve the suitable linewidth that fit with the concentrator dimensions. In **Fig. S2**, the plots represent the evolution of the linewidth as a function of laser power when varying the scan speed. The linewidth slightly increases with the power, and it decreases when increasing the scan speed. Width variation from *500 nm* down to *75 nm* were obtained. Based on these experimental results for this homemade photoresist, the best operating point was found at a scan speed of *75 µm/s* for a laser power in the *6-7 mW* range. In order to obtain experimental dimensions very similar to the adopted design, these conditions were used to implement the polymer concentrators, as mentioned in the accompanying letter. We note that higher speeds (not shown here) were also tested. However, while higher speeds improve the spatial resolution, it typically results in poor mechanical resistance of the polymer concentrators. Further investigation of the achievable spatial resolution with such photoresist will be the focus of a forthcoming paper.

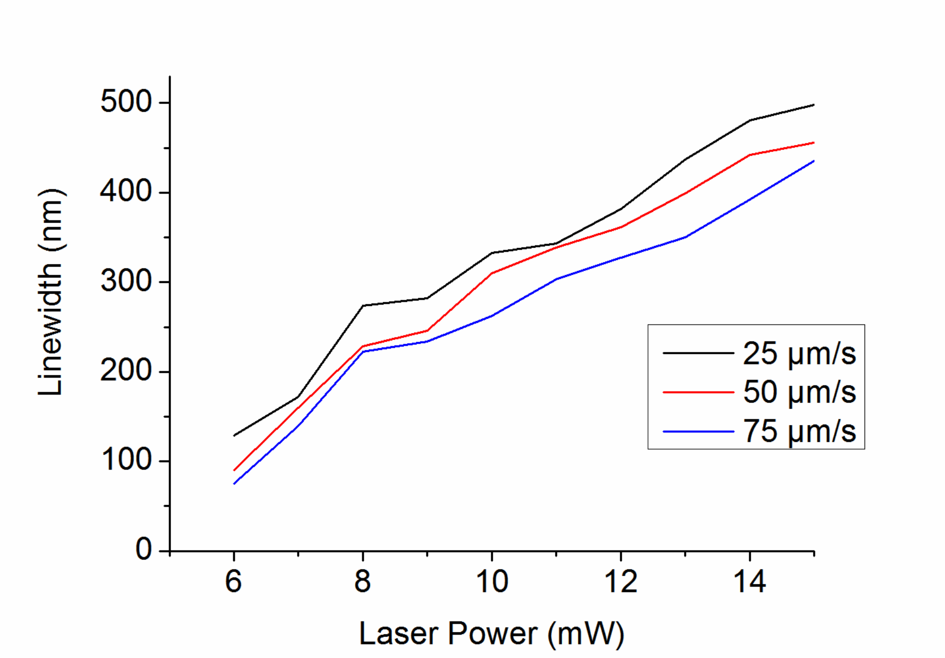

Fig. S4: Evolution of the polymer linewidth as a function of the laser intensity for different writing speeds.
